# Translational Model of Cortical Premotor-Motor Networks

**DOI:** 10.1093/cercor/bhab369

**Published:** 2021-10-23

**Authors:** Svenja L Kreis, Heiko J Luhmann, Dumitru Ciolac, Sergiu Groppa, Muthuraman Muthuraman

**Affiliations:** Institute of Physiology, University Medical Center of the Johannes Gutenberg University Mainz, Mainz D-55128, Germany; Institute of Physiology, University Medical Center of the Johannes Gutenberg University Mainz, Mainz D-55128, Germany; Section of Movement Disorders and Neurostimulation, Biomedical Statistics and Multimodal Signal Processing Unit, Department of Neurology, University Medical Center of the Johannes Gutenberg University Mainz, Mainz D-55131, Germany; Nicolae Testemitanu State University of Medicine and Pharmacy, Chisinau MD-2001, Republic of Moldova; Section of Movement Disorders and Neurostimulation, Biomedical Statistics and Multimodal Signal Processing Unit, Department of Neurology, University Medical Center of the Johannes Gutenberg University Mainz, Mainz D-55131, Germany; Section of Movement Disorders and Neurostimulation, Biomedical Statistics and Multimodal Signal Processing Unit, Department of Neurology, University Medical Center of the Johannes Gutenberg University Mainz, Mainz D-55131, Germany

**Keywords:** functional connectivity, motor control, physiological oscillations, premotor cortex, primary motor cortex, translational study

## Abstract

Deciphering the physiological patterns of motor network connectivity is a prerequisite to elucidate aberrant oscillatory transformations and elaborate robust translational models of movement disorders. In the proposed translational approach, we studied the connectivity between premotor (PMC) and primary motor cortex (M1) by recording high-density electroencephalography in humans and between caudal (CFA) and rostral forelimb (RFA) areas by recording multi-site extracellular activity in mice to obtain spectral power, functional and effective connectivity. We identified a significantly higher spectral power in β- and γ-bands in M1compared to PMC and similarly in mice CFA layers (L) 2/3 and 5 compared to RFA. We found a strong functional β-band connectivity between PMC and M1 in humans and between CFA L6 and RFA L5 in mice. We observed that in both humans and mice the direction of information flow mediated by β- and γ-band oscillations was predominantly from PMC toward M1 and from RFA to CFA, respectively. Combining spectral power, functional and effective connectivity, we revealed clear similarities between human PMC-M1 connections and mice RFA-CFA network. We propose that reciprocal connectivity of mice RFA-CFA circuitry presents a suitable model for analysis of motor control and physiological PMC-M1 functioning or pathological transformations within this network.

## Introduction

Movement disorders such as Parkinson’s disease (PD), tremor, or dystonia are common neurological diseases associated with substantial disability for patients. These conditions are characterized by motor symptoms including bradykinesia, tremor, rigidity, or postural instability that markedly impair the patient’s mobility and life quality (Bloem et al. 2021; Muthuraman et al. 2015). Impaired movement control emerges from aberrant functioning of motor network circuitries (Muthuraman, Koirala, et al. 2018; Muthuraman, Raethjen, et al. 2018). However, how these abnormal patterns arise and disrupt neural networks at the cortical level is largely unknown. Therefore, a more in-depth investigation of connectivity measurements and the development of novel robust models is essential for advancing treatment opportunities for movement disorders. The very first step to decipher these abnormal activity patterns would be the development detailed framework of the physiological connectivity within the premotor-motor network.

In humans, the function and connectivity between the primary motor cortex (M1) and premotor cortex (PMC) have been addressed in several studies (Johansen-Berg et al. 2004; Groppa et al. 2012; Groppa et al. 2012). The M1 cortex encodes the force, direction, speed, and magnitude of voluntary movements (Purves 2001). The PMC encodes motor action instructions associated with a symbolic cue, localization of external stimuli, and movement dynamics (Solopchuk et al. 2016). The interaction between the PMC and the M1 is essential for linking external stimuli (e.g., visual information) to goal-directed movements (Münchau et al. 2002). The PMC has strong anatomical connections with M1 and is known to modulate neuronal activity in the M1 cortex (Chouinard and Paus 2006; Groppa, Schlaak, et al. 2012; Groppa, Werner-Petroll, et al. 2012). We described by the aid of multifocal stimulation a short latency facilitatory pathway connecting the left dorsal PMC and the M1 hand area in humans (Groppa, Schlaak, et al. 2012; Groppa, Werner-Petroll, et al. 2012). Abnormal oscillatory responses and altered functional connectivity within the motor network circuits, comprising the PMC and M1 among others, have been evidenced in PD and dystonia patients (Poston and Eidelberg 2012; Carmona et al. 2017).

In contrast to humans and non-human primates, available data on the organization and connectivity of different areas of the motor cortex in mice is scarce (Tennant et al. 2011). Mouse models offer the possibility for a variety of genetic, pharmacological, or optogenetic manipulations as well as the opportunity to analyze pathophysiological alterations on a cell- and cortical layer-specific level, representing an extremely valuable tool for research in the field of human movement disorders (Iderberg et al. 2012; Morin et al. 2014; Baaske et al. 2020). Therefore, it is essential to gain more detailed information on the connectivity of the PMC-M1 network in mice.

Intracortical microstimulation studies have shown that the forelimb representation of the mouse motor cortex is divided into at least two functionally distinct areas—the rostral forelimb area (RFA) and the caudal forelimb area (CFA) (Rouiller et al. 1993; Tennant et al. 2011). The CFA is thought to be the equivalent of the M1 cortex in the human brain and the RFA could be regarded as the homolog of the PMC (Komiyama et al. 2010; Sul et al. 2011; Brown and Teskey 2014).

Striking similarities in the effects of stimulation of the RFA and CFA on motor performance suggest that both areas may be part of a highly integrated computing unit (Saiki et al. 2014; Morandell and Huber 2017). Both areas are activated during skilled forelimb movements and are equally essential for the execution of skilled limb movements (Brown and Teskey 2014; Guo et al. 2015; Galiñanes et al. 2018). Currently, it is still unclear how the RFA and CFA are functionally interconnected on the level of layer-specific circuits. The frequency-specific connectivity within the mouse motor cortex, especially between RFA and CFA, is poorly understood. In contrast, in humans, neuronal synchronization in the gamma frequency band is associated with neural coding and plays a key role in the processing of information in task-specific neuronal networks (Gray 1999; Buzsáki and Schomburg 2015). Synchronization in the beta band within the motor cortex coordinates the processing of sensorimotor information (Athanasiou et al. 2018) and mediates the preparation and execution of movements (Pavlidou et al. 2014). In addition, electroencephalography (EEG) recordings from PD patients showed that increased oscillatory activity in the beta frequency band network is related to the severity of motor impairment (Brown et al. 2001; Tinkhauser et al. 2017; Gonzalez-Escamilla et al. 2020). Therefore, we focused our analysis on the activity in the beta and gamma frequency bands in both mice and humans.

We developed a translational analytical framework to obtain information on functional and effective connectivity between layer (L) 2/3, 5, and 6 of the RFA and CFA in male C57BL6/*N* mice.

Functional connectivity is defined as statistical dependencies among remote neurophysiological events. In this study we use coherence analysis to analyze functional connectivity. In contrast to functional connectivity, effective connectivity describes the direction of information flow between two regions. We have measured the effective connectivity between RFA and CFA in mice, PMC and M1 in humans by applying time-resolved partial directed coherence (TPDC; Muthuraman, Raethjen, et al. 2018).

Using a combination of these methods on data recorded in humans as well as in mice, we were able to build a conceptual framework of the human PMC-M1 connectivity and the mouse RFA-CFA network. We were able to compare the functional and effective connectivity in the premotor-motor network of humans to the findings on the RFA-CFA network in mice. Within the statistical approach of predictive modeling, we applied structural equation models (SEM) to the estimated functional parameters namely spectral power, coherence, and effective connectivity, and selected the models that best predicted spike units at the different topological layers for both regions. We aimed to validate a causal relation between the spike units and connectivity in these two motor regions in the mouse motor cortex.

In this study, we addressed the following questions:

1. Can the human PMC and M1 areas be distinguished based on their spectral power and how these two motor cortices are functionally interconnected?
2. Are the two mouse motor cortex areas, the RFA and CFA, electrophysiologically distinct areas?
3. What are the features of functional and effective connectivity between the RFA and CFA?
4. Can the connectivity of the RFA-CFA network be used as a valid model for the human PMC-M1 network?

By answering these questions, we aimed to validate the RFA-CFA network in mice as a suitable model for research in human movement disorders.

## Methods

### Healthy Human Subjects

In this study, 34 healthy subjects (age: 58 ± 9 years; nine females; 29 right-handed) were recruited. Participants were seated in a comfortable chair in a slightly reclined position. Both forearms were supported to the wrists by firm armrests. The EEG recordings were performed at resting state of 10 min with eyes closed.

### E‌EG Recordings and MRI

The EEG was recorded with a high-density 256-channel recording system (Magstim EGI), with Cz as reference at a sampling rate of 5000 Hz. Data were analyzed offline. All subjects underwent MRI using a 3 T MRI scanner (Siemens TrioTim) with a 32-channel head coil. This included whole-brain high-resolution T_1_-weighted images using the MPRAGE (magnetization-prepared 180° radio-frequency pulses and rapid gradient-echo) sequence with repetition time = 1900 ms, echo time = 2.52 ms, flip angle = 9, and voxel resolution of 1 × 1 × 1 mm^3^.

### Processing of EEG Data and Source Analysis

Initially, EEG data were re-referenced to the common grand average reference of all channels. The raw data were low-pass filtered (fourth-order Butterworth filter; cut-off frequency: 500 Hz) to avoid aliasing, followed by high-pass filtering at 0.5 Hz. Then, data were subjected to independent component analysis (FastICA) to remove components related to muscle activity, eye blinks, eye movements, and line noise-artifacts. On average, 9 of 256 components [9 ± 3.4, mean ± standard deviation (SD) were rejected (eye artifact: 5 ± 0.68; line noise: 2 ± 0.34; muscle artifacts: 2 ± 1.21]. Residual muscle artifacts were visually inspected, removed, and interpolated with the cubic interpolation method. Each recording was segmented into several 1-s epochs (L = 5000). Depending on the length (N) of the recording and the quality of the data, 240–260 1-s epochs (M) were used for analysis, such that N = L^*^M. To solve the EEG forward problem in source reconstruction, we estimated the volume conduction model and the lead-field matrix (LFM) containing information about the geometry and conductivity with the finite-element method (FEM) (Wolters et al. 2007). Skin, skull, CSF, and gray and white matter surfaces were extracted from the individual anatomical T1-MPRAGE scans, and individual electrode locations were used. A complete protocol has been described previously (Muthuraman et al. 2010, 2012). A full outline of the beamformer linear constrained minimum variance spatial filter is given elsewhere (van Veen et al. 1997; Muthuraman,Raethjen, et al. 2018). The output of the beamformer at a voxel in the brain can be defined as a weighted sum of the output of all EEG channels. The frequency components and their linear interactions are represented as a cross-spectral density (CSD) matrix. To visualize power at a given frequency range, we used a linear transformation based on a constrained optimization problem, which acts as a spatial filter (van Veen et al. 1997). The spatial filter assigned a specific value of power to each voxel. For a given source the beamformer weights for a location of interest are determined by the data covariance matrix and the LFM. Voxel size was 2 mm, resulting in 6676 voxels covering the entire brain.

The human M1 and PMC ROI’s were defined based on the beamformer linear constrained minimum variance spatial filter. The power at any given location in the brain can be computed using a linear transformation which in our case is the spatial filter. The spatial filter relates the underlying neural activity to the electromagnetic field in the surface. The neural activity is modeled as a current dipole or sum of current dipoles. The main aim of the LCMV method is to design a bank of spatial filters that attenuates signals from other locations and allows only signals generated from a particular location in the brain.

**Figure 1:**
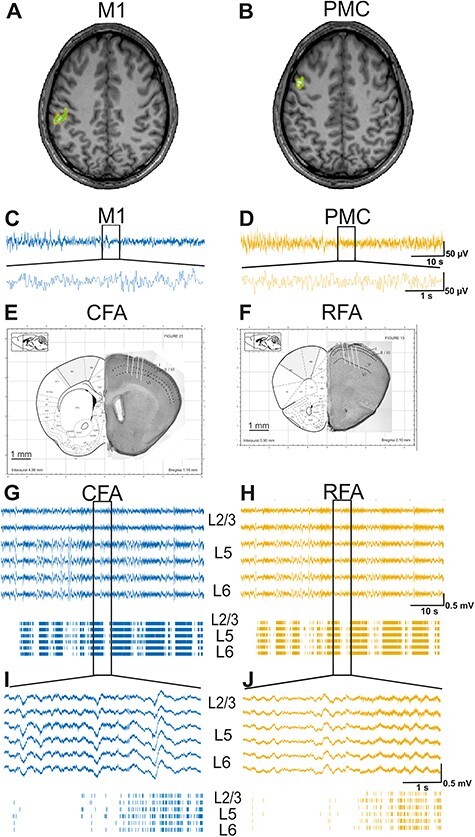
Reconstruction of recording area and spontaneous activity recorded in human and mouse motor cortex. (*A*, *B*) Source grand average from all healthy human subjects in the β-frequency band in the M1 (*A*) axial slices and the PMC (*B*) is shown; (*C*, *D*) simultaneous recordings of spontaneous EEG signal in M1 (*C*, blue) and PMC (*D*, yellow). Representation of a 60-s segment of raw data (on top) and magnification of a 5-s segment (below); (*E*, *F*) Localization and configuration of the MEAs for simultaneous recordings in the CFA (*E*) and RFA (*F*) of an adult C57Bl6-N mouse in vivo. Left panels of (*A*) and (*B*) are adapted from Paxinos and Franklin (2005), shaded areas indicating the CFA and RFA (Tennant et al. 2011); right panels of (*A*) and (*B*) show Nissl staining; black dashed lines indicate layer boundaries; I–VI indicate the layer number; white dashed lines indicate tracks of the MEAs; (*G*–*J*) simultaneous recordings of spontaneous activity from layers L2/3 (first and second trace), L5 (third, fourth trace), and L6 (fifth, sixth trace) of CFA (*G*, blue) and RFA (*H*, yellow). Representative 60-s segments of LFPs (*G*, and *H*) and MUA (bottom panel) were recorded simultaneously in RFA and CFA (top). Lower traces show a 5-s segment at higher temporal resolution (*I*, *J*).

For the beta (14–30 Hz) and gamma (31–100 Hz) bands, a within-subject surrogate analysis was used to define the significance level in order to identify activated voxels in the M1 and PMC. Their activity was then extracted from the source space for further analysis. The source grand average from healthy subjects in the beta frequency for the M1 and PMC regions is shown in Figure 1*A*,*B*.

## Rodents

### Surgical Preparation and Electrophysiological Recordings

For acute recordings, mice (age: 2–4 months, 24–32 g) were anesthetized with 3% isoflurane and maintained under anesthesia with urethane (1 mg/g, i.p) and 1–2% isoflurane during the surgery. Additionally, lidocaine gel was applied to the skin in the area of the incision before the surgery. The sufficient depth of anesthesia for the operation (surgical tolerance stage) was determined pre-operatively and intra-operatively by the absence of the inter-toe reflex and the eyelid reflex. Eyes were covered with eye ointment. The mice were placed on a heating pad to prevent hypothermia during surgical preparation and electrophysiological recording. After the mice were fixated with a custom-made head holder and a suitable frame, a silver wire was implanted into the cerebellum as a reference electrode. Subsequently, a craniotomy was performed over the front paw representation of the motor cortex without damaging the dura. During recordings, breathing rate and presence or absence of the pinch toe reflex were used as indicators of the anesthetic depth during electrophysiological recordings.

We performed extracellular microelectrode array (MEA) recordings in all layers of the RFA and CFA. For the recording, 32-channel MEAs with a 4-shank configuration (A4x8-A32, Neuronexus; 200-μm distance between the shanks, 100-μm vertical distance between contacts) were slowly inserted into the RFA (2.5-mm AP, 1.2-mm ML) and CFA (0.5-mm AP, 0.8-mm ML) to a maximum depth of 0.8–1 mm (coordinates were chosen according to Tennant et al. 2011). After stabilization of the neuronal signal, ~30 min after electrode insertion, extracellular signals were recorded for 10 min at 20 kHz with the ME2100 system (Multi Channel Systems).

### Histology

The recording electrodes were coated with a fluorescent lipophilic dye (DiI, D282, Invitrogen). After the experiment, animals were deeply anesthetized with xylazine hydrochloride (30 mg/kg) and transcranially perfused with cold 0.1-M phosphate buffer, followed by 4% paraformaldehyde (PFA). The brain was removed and fixed in 4% PFA at 4 °C overnight. Histological brain slices (200-μm thickness) were prepared using a freezing microtome (Leica) and analyzed for traces of fluorescent DiI in order to identify the exact location and depth of the electrodes. Cresyl violet staining was performed to identify cortical layers (Fig. 1*E*,*F*).

### Data Analysis

Data analysis was performed using the FieldTrip toolbox (Oostenveld et al. 2011) and custom programs written in MATLAB (version 2019b; Mathworks). The full sampling rate of 20 kHz was used to analyze local field potential (LFP), spike detection, sorting, and single unit (SU) analysis. Spectral power, coherence, and TPDC results were split into different frequency bands (delta = 0–3 Hz, theta = 4–7 Hz, alpha = 8–15 Hz, beta = 16–31 Hz, low gamma = 32–49 Hz, medium gamma = 51–75 Hz, and high gamma = 76–100 Hz).

### LFP Power

The LFP power was calculated using multitaper frequency transformation. First, the recorded signals were high-pass filtered at 0.1 Hz and low-pass filtered at 250 Hz. Additionally, 50-, 100-, and 150-Hz notch filtering was applied to reduce 50-Hz noise. The spectral power was calculated on 50-ms segments from 0.1 to 100 Hz with a step size of 0.25 Hz. The resulting spectra were sorted according to the layer of recording, pooled for all animals, and separated into different frequency bands.

### Functional Connectivity Analysis

To analyze the functional connectivity between layers 2/3, 5, and 6 of RFA and CFA, we calculated the LFP coherence between every pair of recording electrodes for each animal. First, the raw signal was bandpass filtered at 0.5–250 Hz. The spectral coherence was calculated for frequencies between 0.5 and 100 Hz with a step size of 0.5 Hz. The resulting coherence was split into layers and frequency bands and pooled for all animals. The significance was tested by a within-subject surrogate analysis, in which the surrogates were estimated by a Monte Carlo random permutation 100 times shuffling of 500-ms segments within each subject. The surrogates possess the same mean, variance, and histogram distribution as the original signal, but any temporal structure is destroyed.

### Effective Connectivity

#### Spike Sorting

Spike detection and sorting was performed using WaveClus 3 (Quian Quiroga et al. 2018). The continuously recorded wide-band signals were high-pass filtered (0.3–3 kHz). Spike detection was performed in each channel independently using amplitude thresholding in the negative range. The threshold level was set independently for each channel as 5 times the SD of the signal. Peaks that exceeded 30 SD of the noise level were considered to be an artifact and excluded. When a threshold crossing was detected, all sampled amplitude values from this channel in the time range from −0.5 to +3 ms relative to the negative peak were extracted. Feature extraction uses a selection of wavelet coefficients chosen with a Kolmogorov Smirnov test of Normality (Quiroga et al. 2004). Super-paramagnetic clustering (SPC) was used to define SU clusters. The SPC is based on simulated interactions between each data point and its K-nearest neighbors; the method is implemented as a Monte Carlo iteration of a Potts model (for detailed explanation see Quiroga et al. 2004). After automated clustering, outliers were rejected from the SUs using the FieldTrip toolbox. Finally, the quality of the SU was analyzed using the MLIB toolbox (Stüttgen 2020). Incomplete separated or unstable SUs were excluded from the analysis.

#### Time-Resolved Partial Directed Coherence

Coherence only shows whether and to what extent two regions are interconnected but does not indicate the direction of information flow between the analyzed areas. Therefore, we additionally used the time–frequency causality of TPDC, which allows the analysis of not only certain frequency bands but also the time dynamics and direction of causality at this frequency. Significance was tested by means of time-reversal technique (Haufe et al. 2013). We chose one channel per area, layer, and animal, based on the highest theta power to TPDC analysis. Thus, we analyzed the connectivity between three channels in the RFA and three channels from the CFA. The LFP data were downsampled to 200 Hz before applying the TPDC analysis (Muthuraman, Raethjen, et al. 2018).

### Statistical Analysis

Statistical analysis was performed in MATLAB (version R2019a, Mathworks). We used an explorative approach to investigate the region-, layer-, frequency-, and direction-specificity of the neuronal signals. Linear mixed-effects models (LMM) were used to identify significant effects. For the LMM results that showed significant effects of specific factors, two-sampled *t*-tests were performed. All post-hoc tests have been Bonferroni corrected to account for multiple comparisons (e.g., different layer combinations or different frequency bands).

### Structural Equation Modeling

The SEM analysis was performed in the SEM toolbox for MATLAB (version R13a, Mathworks). The SEM represents a complex analytical tool that can determine the causal relationships between the variables in a model-based approach. We applied several models with the aim to predict the causal relations between the effective connectivity and spike units. Initial models were built with one mediator and step-by-step increased the complexity with two mediators to attain a significant causal relation between the input and output. We describe the structure of the best two models as follows: in the first model, the relationship between the TPDC connectivity (i.e., RFA L5 to CFA L2/3) and spike SU (separately for RFA L5 and CFA L2/3) in the beta frequency. The mediator one was coherence between RFA L5 and L2/3 and the second mediator was separately power at RFA L5 and CFA L2/3. In the second model, the same structure was used for the high gamma frequency. We tested the same model structure for all the analyzed frequencies with all pair-wise layer combinations.

We employed the Maximum Likelihood method of estimation to fit the models. We used the Root Mean Square Error of Approximation (RMSEA) index to improve the precision of the model without increasing the bias (Kelley and Lai 2011). The RMSEA index estimates lack of fit in a model compared with a perfect model and therefore should be low. The RMSEA index for all models was below 0.05, implying a very good fit of the models. In all models, the Invariant under a Constant Scaling (ICS) and ICS factor criteria should be close to zero, indicating that models were appropriate for analysis. Finally, by Akaike Information Criterion (AIC), the quality of each model relative to other models was estimated, with smaller values signifying a better fit of the model. The obtained AIC comparing the models varied between 0.01 and 0.03 (good fit of the models). The strength of associations between the variables in the models was quantified by standardized coefficients (s), ranging from 0 (no association) to 1 (very strong association). *P*-values of *α* < 0.05 were considered statistically significant.

### Study Approval

The study protocol was approved by the local ethics committee of the Universitätsmedizin Mainz (#23177–07/G 14–1-080). All subjects provided written informed consent before the procedure. Animal experiments followed the European and German national regulations (European Communities Council Directive, 86/609/ECC). In total, 16 *C57BL6/N* male mice were used.

## Results

### Properties of EEG Activity in the Human PMC and M1

First, we addressed the question of whether EEG recordings from the human PMC and M1 differ in their spectral power and how these two motor cortices are functionally interconnected in healthy human subjects. To do so, we recorded and analyzed EEG signals from the PMC and M1 regions from 34 healthy human subjects (Fig. 1*A*–*D*).

**Figure 2:**
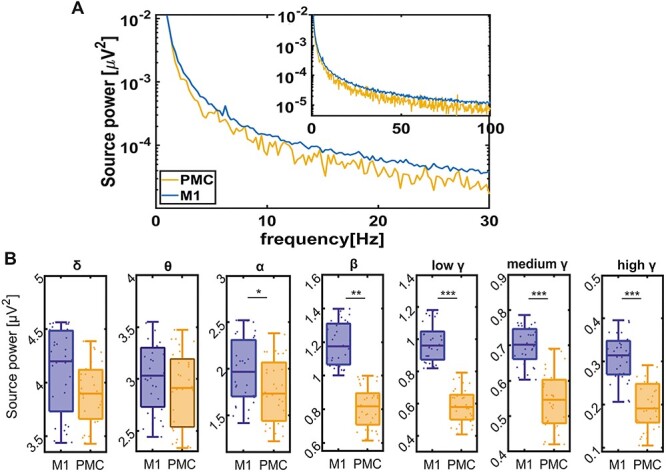
Spectral power in M1 and PMC of human subjects. (*A*) Spectral power of spontaneous EEG recordings in M1 (blue) and PMC (yellow). (*B*) Boxplots of the spectral power were separated into seven frequency bands for EEG activity recorded in M1 (blue) and PMC (yellow). Note the differences in scale between the frequency bands; all plots show the mean and standard deviation of 34 healthy human subjects, each individual is represented by a dot; ^*^ represents *P* < 0.05, ^*^^*^P < 0.001, ^*^^*^^*^P < 0.0001.

We calculated the spectral power and then divided the results into frequency bands (Fig. 2*A*) The EEG power was higher in M1 as compared with PMC and this difference was significant for the α, β, and low-to-high γ-bands (all *P* < 0.05) (Fig. 2*B*).

### Premotor-Motor Connectivity in Human Subjects

**Figure 3:**
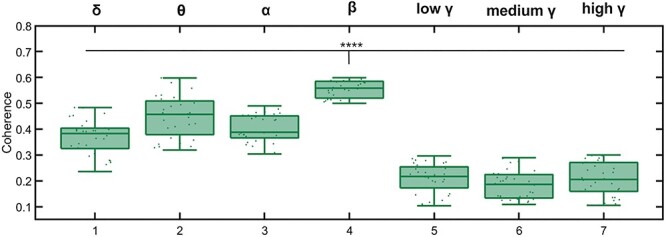
Coherence between spontaneous EEG recordings in M1 and PMC. The coherence in the β-band was significantly higher when compared with the coherence in all other bands (^*^^*^^*^^*^ represents *P* < 10–9). All boxplots show the mean and standard deviation of 34 healthy human subjects; each individual is represented by a dot.

Next, we analyzed the functional connectivity between M1 and PMC. We found significant coherence between M1 and PMC for all frequency bands (Fig. 3). The coherence in the β frequency band was significantly higher (*P* < 10^−9^) as compared with the coherence in all other frequency bands.

These results show that PMC and M1 are highly interconnected in a frequency-specific manner.

**Figure 4:**
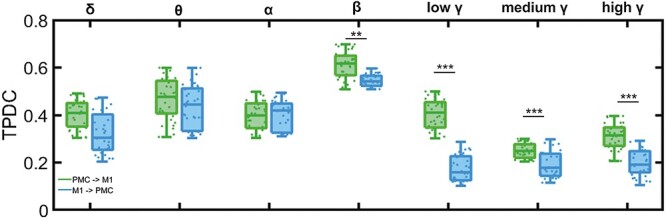
The TPDC connectivity between PMC and M1 of human subjects. Boxplots of TPDC separated into seven frequency bands for the direction PMC → M1 (green) and M1 → PMC (blue); all plots show the mean and standard deviation of 34 healthy human subjects; each individual is represented by a dot; ^*^^*^ represents *P* < 0.001, ^*^^*^^*^P < 0.0001.

To analyze this connectivity in more detail, we calculated the mean TPDC between the two areas. This method is used to gain insights into the direction of information flows between PMC and M1 (Fig. 4). We found that the TPDC is generally higher for the direction from PMC toward M1. This difference in direction of information flow was significant for the β and low-to-high γ bands. The TPDC was highest in the β frequency band for the direction from the PMC toward the M1.

These data demonstrate a strong bidirectional and frequency-specific functional connectivity between PMC and M1 in humans.

### Premotor-Motor Network in the Mouse Motor Cortex

In order to validate the mouse motor cortex areas, the RFA and CFA, as a suitable model for the human premotor-motor network, we simultaneously recorded spontaneous neuronal activity in layers 2/3, 5, and 6 of RFA and CFA in lightly anesthetized mice in vivo.

### Spontaneous Activity in Mouse Motor Cortex

First, we addressed the question of whether RFA and CFA show differences in their spontaneous neuronal activity. We recorded 10 min of spontaneous activity from layers 2/3, 5, and 6 of RFA and CFA. The resulting data were analyzed and illustrated as LFP (0.1–250 Hz) and multi-unit activity (MUA; 0.3–3 kHz). A 60-s segment of LFP raw signal and the corresponding MUA activity is shown in Figure 1*G*,*H* and below a 5-s segment in higher magnification (Fig. 1*I*,*J*). Both LFP and MUA from RFA and CFA contain periods of high activity separated by segments with lower activity. The LFP signal showed strong similarities between the layers. This similarity between the layers is also visible in the MUA of RFA and CFA.

To further analyze the LPF signals, we calculated the spectral power for layers 2/3, 5, and 6 of RFA and CFA. As in the analysis of human data, the resulting power spectrum was then divided into frequency bands for a more detailed analysis.

**Figure 5:**
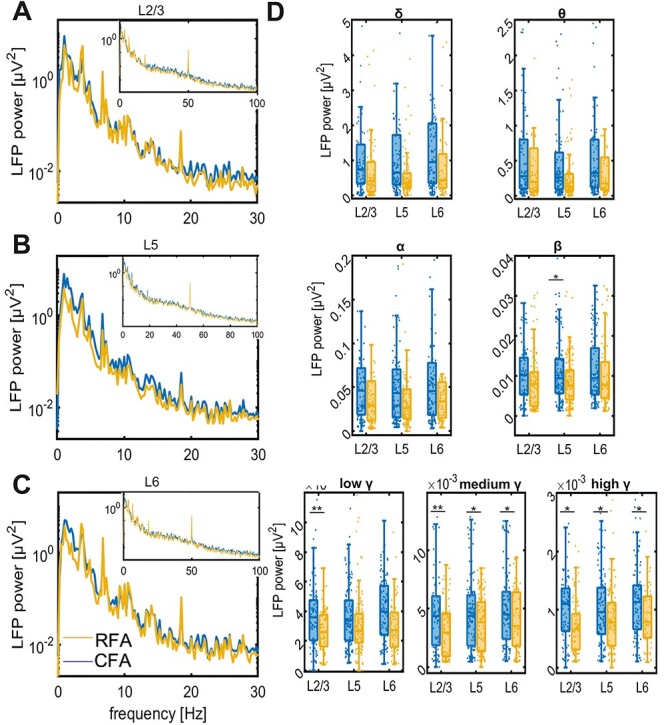
Spectral power in mice CFA and RFA. Spectral power of spontaneous LFP activity recorded in CFA (blue) and RFA (yellow). The LFP power spectrum for L2/3 (*A*), L5 (*B*), and L6 (*C*) for 0–30 Hz of CFA (blue) and RFA (yellow). Inset figures show the corresponding spectrum for 0–100 Hz. (*D*) Boxplots of the spectral power separated into seven frequency bands averaged across 16 mice. Each analyzed channel is represented by a dot; scale is different in every boxplot to visualize the differences between RFA and CFA. ^*^ represents *P* < 0.05, ^*^^*^P < 0.001.

The LFPs in all layers of both motor regions showed a high power at lower frequencies (Fig. 5). Similar to the human data on PMC and M1, the strongest power was observed in the delta frequency band. Alike to the results in the human motor cortex (Fig. 2), we found that the LFP power in the β- and low-to-high γ-bands was significantly higher in the CFA as compared with the RFA (Fig. 5, Supplementary Table 1). These data indicate that the RFA and the CFA are topologically separate areas.

For the subsequent analysis of spontaneous activity, we used threshold-based clustering to a total of 436 isolated SU from 16 animals. The mean SU firing rate did not show significant differences in the firing rates between the different layers in RFA and CFA (Supplementary Table 2). The number of SU was higher in CFA (*n* = 302) as compared with RFA (*n* = 134). We detected a much higher number of SU in L6 of CFA (154) as compared with RFA and CFA L 2/3.

### Functional Connectivity between RFA and CFA

**Figure 6:**
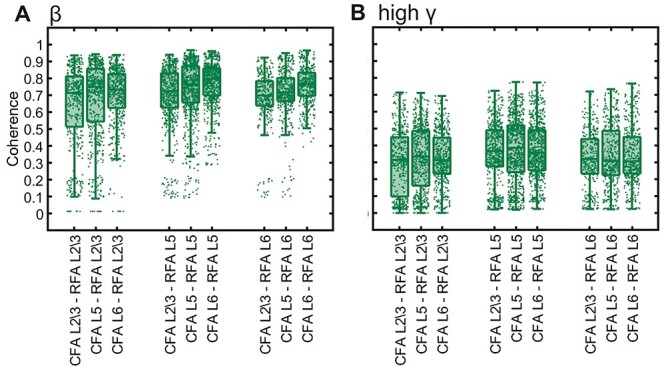
Coherence in mice CFA and RFA layers. Comparison of LFP coherence between layers 2/3, 5, and 6 of RFA and CFA. Boxplots for the beta (*A*) and high gamma (*B*) frequency bands averaged across 16 mice; each analyzed channel is represented by a dot.

Next, we addressed the question of whether and how the two motor cortical areas, RFA and CFA, are functionally interconnected in a frequency- and layer-specific manner. We analyzed the LFP coherence of spontaneous activity recorded in layers 2/3, 5, and 6 from RFA and CFA applying identical analysis tools as for the analysis of the human EEG data. Then, we averaged the resulting coherence across all 16 animals (Fig. 6). Our results show significant coherence between RFA and CFA for all layer combinations and frequency bands. The highest coherence was observed in the β-frequency band between CFA layer 6 and RFA layer 5 (0.7670 ± 0.1293, Supplementary Table 3). The coherence in the β-band was significantly higher as compared with the coherence in all other frequency bands (*P* < 10^−12^); this is in line with our results on the connectivity between PMC and M1 in humans (Fig. 3).

We used LMM to test for significant differences in LFP coherence between the layer combinations of RFA and CFA. We found significant differences in the β-frequency band between the coherence of RFA L5 and L6 to all CFA layers (RFA L5: *P* = 3.85e^−08^; RFA L6: *P* = 9.52e^−05^) and between CFA L5 and L6 to all RFA layers on the resulting coherence (CFA L5: *P* = 0.0006, CFA L6: *P* = 8.06e^−08^).

Comparable results were found in all frequency bands (Supplementary Table 3). The coherence between RFA L5 and L6 to CFA L6 was significantly higher than the coherence for any other layer-combination in the δ- and θ-frequency bands. These results indicate that both cortical regions are functionally coupled in a layer-specific manner in all frequency bands.

### Effective Connectivity between RFA and CFA

**Figure 7:**
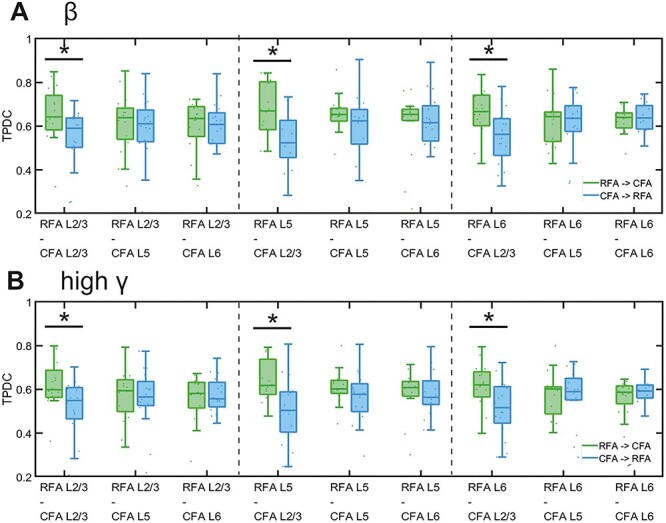
The TPDC connectivity between mice RFA and CFA. Layer-specific directionality of information flow between layers 2/3, 5, and 6 of RFA and CFA in β (*A*) and high γ (*B*) bands. Boxplots of TPDC for the directions RFA → CFA (green) and CFA → RFA (blue); averaged across 16 animals. ^*^ indicates significant effects of transmission direction as identified by LMM.

Next, we addressed the question of whether we can identify a bidirectional connectivity between RFA and CFA using LFP data. In addition to that, we asked whether we can identify differences in the direction of information transmission. Therefore, we performed TPDC to further quantify the direction of connectivity between RFA and CFA based on the recorded LFP. We calculated the mean TPDC for each layer combination and frequency band for the direction from RFA toward CFA as well as vice versa (Fig. 7). We found that the TPDC is generally slightly higher for the direction from RFA toward CFA. We used LMM to test for significant effects of the different layers from RFA and CFA as well as for a possible effect of the direction of interaction between the two areas. The LMM confirmed that the flow of information is significantly higher from all RFA layers toward CFA L2/3 in all frequency bands (Fig. 7). Significant differences between the TPDC values for the direction from and toward CFA L2/3 were confirmed using additional two-sample *t*-tests; the resulting *P*-values are given in Supplementary Table 4. We could show that the flow of information from RFA L6 to CFA L2/3 is significantly higher in all frequency bands, as compared with the opposite direction. The input from RFA L5 to CFA L2/3 was significantly higher than the output from CFA L2/3 to RFA L5 in the δ (*P* = 0.0231), θ (*P* = 0.0332), low γ (*P* = 0.0465), medium γ (*P* = 0.0399), and high γ (*P* = 0.0388) frequency bands. The flow of information from RFA L2/3 to CFA L2/3 was significantly higher in the θ (*P* = 0.0203), α (*P* = 0.0220), β (*P* = 0.0230), and high γ (*P* = 0.0166) bands (Supplementary Table 5). This is in line with our findings on the direction of connectivity from the PMC toward M1 in the human motor cortex (Fig. 4).

These data confirmed a strong bidirectional connectivity between all RFA and CFA layers. The transmission of information was stronger from RFA toward CFA, especially for the combination of all RFA layers toward CFA L2/3.

### Structural Equation Modeling

Finally, we addressed the question of whether the SU activity can be inferred based on the spectral measures of LFPs, which were recorded from the mouse RFA and CFA.

**Figure 8:**
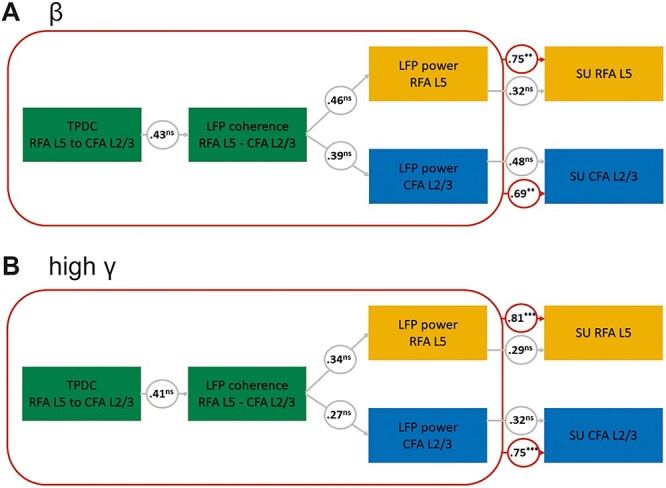
Structural equation modeling (SEM). Results from the SEM analysis for the β-frequency band (*A*) and the high γ-frequency band (*B*). Numbers in circles represent standardized coefficients; gray lines and circles indicate the relationship between the individual parameters; red lines and circles indicate significant coefficients of the overall performance of the model; ns stands for not significant, ^*^^*^ indicates *P* < 0.001, ^*^^*^^*^ indicates *P* < 0.0001.

The obtained fit indices in the SEM analysis implied a good fit of the constructed models to the observed data, providing robust causal relations between the parameters. In the first model, we identified significant causal relations between the TPDC from RFA L5 to CFA L2/3 as input and coherence between the RFA L5 to L2/3 (as the first mediator) and power separately at RFA L5 and L2/3 (as the second mediator) and SU in the RFA L5 (*s* = 0.75, *P* < 0.01) and CFA L2/3 (*s* = 0.69, *P* < 0.01) in the beta frequency (Fig. 8*A*). Moreover, in the high gamma frequency with the same model structure, we were able to establish similar significant causal relations between the TPDC and SU in the RFA L5 (*s* = 0.81, *P* < 0.001) and CFA L2/3 (*s* = 0.75, *P* < 0.001) (Fig. 8*B*).

## Discussion

In this study, we applied a combination of spectral power, functional, and effective connectivity analysis on spontaneous neuronal activity recorded from PMC and M1 areas of healthy human subjects as well as from RFA and CFA of wild-type mice. Our data revealed strong similarities between human PMC-M1 functional connections and the RFA-CFA network in the mouse. Our results attested significantly different spectral power characteristics in the human PMC and M1 and strong functional interconnections between these areas. The information flow predominantly followed the direction from PMC toward M1 in the β and low-to-high γ frequency bands. Similar to the results obtained in humans, the mice data demonstrated that the two motor areas, RFA and CFA, can be topologically characterized by electrophysiological proxies. Alike PMC and M1, RFA and CFA are functionally strongly interconnected and show layer- and frequency-specific patterns of connectivity. The designed SEM model demonstrated significant causal relations between LFP power, coherence, TPDC, and SU activity in RFA L5 to CFA L2/3. Major differences in oscillatory activity between the premotor and primary motor areas were found in the β and high γ-frequency bands in both the human and mouse motor cortex network. This indicates that these frequency bands can be used as biomarkers for dysfunctions in the premotor-motor network.

### The Resting-State PMC to M1 Connectivity in Healthy Human Subjects

The significantly higher spectral power across frequencies in the M1 compared with the PMC was to be expected as the PMC is known to play an essential role in sensorimotor and visiomotor integration during the action control (Chouinard and Paus 2006). We further observed a high coherence between the EEG signals recorded from the human PMC and M1 that was the highest in the β-frequency band. Indeed, synchronization in the β-frequency band was associated with the processing of sensorimotor information during resting state (Engel and Fries 2010; Athanasiou et al. 2018). When we analyzed the direction of information transmission between the PMC and M1 in humans at rest, our results showed a strong bidirectional connectivity between both areas. However, the information flow in β- and low-to-high γ-frequency bands from the PMC toward the M1 was significantly stronger compared with the opposite direction, confirming that the PMC strongly influences neural activity in the M1 (Münchau et al. 2002; Chouinard et al. 2003; Chouinard and Paus 2006).

In our previous work (Tamás et al. 2018) on healthy humans, we analyzed the gamma synchronization at the beginning of both a simple brisk and a combined movement, and during a medium-strength isometric contraction, and showed that the direction of information flow was from the M1 to the PMC during hand movements. In contrast, in this study, we analyzed resting state data without any active motor processing which could explain the difference in connectivity.

### Mouse Motor Cortical Areas RFA and the CFA as a Model for Human Premotor-Motor Network

By employing MEAs, we recorded spontaneous activity simultaneously at multiple sites in layers 2/3, 5, and 6 of RFA and CFA in lightly anesthetized adult C57Bl6-N mice. In addition to the estimation of functional connectivity using LFP coherence, we studied the direction and strength of the effective connectivity by SU cross-correlations and TPDC. Our results demonstrated that RFA and CFA are two functionally distinct cortical areas that are highly interconnected and share common inputs from other brain structures. All layers in both areas showed a strong synchronization during spontaneous activity; however, synchronization was the strongest between RFA L5 and CFA L2/3 in β-and high γ-frequency bands. By analyzing the direction of information transmission with TPDC, we identified the RFA as the moderator of transmission.

These findings do not only contribute to a better understanding of the interactions in the premotor-motor network in mice but also identified strong similarities between the mouse RFA-CFA and the human PMC-M1 networks. This implies that connectivity between the RFA and CFA might be a useful model for the human premotor-motor network. Thereby, this study fosters future research on movement disorders, such as PD or Huntington disease, using mouse models. In our study, we focused on the β- and high γ-frequency bands, since these frequency bands are commonly used as biomarkers in the motor cortex in rodent models for PD (Dupre et al. 2016).

Both the RFA and the CFA revealed the strongest spontaneous activity in the delta and theta frequency range and decrease of spectral power from α- over β- to the γ-frequency band. The power was higher in CFA compared with RFA, being in concordance with our results on spectral power differences between the human PMC and M1. The recorded higher mean firing rate of SU in the RFA compared with the firing rate in the CFA suggests that these two are electrophysiologically distinct areas.

Our data show a strong LFP synchronization between all layer combinations of RFA and CFA in a wide range of frequencies. The highest coherence was observed in the β-band between the RFA L5 and L6, and CFA L6. These results corroborate previous studies showing anatomical connections between all RFA and CFA layers (Rouiller et al. 1993; Hira et al. 2013). Hira et al. (2013) showed extensive anatomical connections between deeper layers of RFA and CFA. Although the mouse data were measured in the anesthetized state, the coherence between RFA and CFA was comparable to the coherence between PMC and M1 in humans, being significantly higher in the β-band as compared with the other frequency bands.

Similar to the human data, we found reciprocal interactions in all pairs of layer combinations between the RFA and CFA, which is consistent with previous studies (Rouiller et al. 1993; Harrison et al. 2012; Hira et al. 2013; Bukhari et al. 2018). Alike in humans, the flow of information was stronger from RFA toward CFA for all layer combinations. This difference in transmission strength was significant in all frequency bands for interactions between all RFA layers to CFA layer 2/3. Taken together, our results indicate that RFA has a strong modulatory influence on the neural activity of CFA and is supported by the findings of Deffeyes et al. (2015), who applied intracortical microstimulation techniques and found that RFA has a strong modulatory effect on CFA activity.

### Model Framework

To detect causal relations between the effective connectivity and spike units, we applied SEM, which is a form of causal modeling that fits networks of constructs to data. Our findings showed a strong association between the effective connectivity between the L2/3 and L5 and SU in beta and high gamma frequency bands. Finally, our model-based approach goes beyond the correlation analysis and demonstrates that SEM can be applied to layer-specific mice data to investigate the causal pathways that mediate the connectivity between the two topologically distinct motor regions.

### Oscillations at β- and γ-Frequencies as Biomarker in the Premotor-Motor Network

Neuronal oscillatory signals at different frequencies are known to coordinate the time-locked information transfer across brain regions and, thereby, promote physiological brain activity for coordination of movements or cognitive functions. It is known that alterations in the amplitude or coherence of gamma-band oscillations are related to a variety of processes such as attention, multisensory and sensorimotor integration, and movement preparation (Engel and Fries 2010). Synchronization in the β-frequency band in the motor cortex is associated with the maintenance of the current body position without further movement (Engel and Fries 2010). We found significant differences in power, functional, and effective connectivity in the β- and high γ-frequency bands both in the RFA-CFA network in mice and the human PMC-M1 connections. These results demonstrate that β- and high γ-frequency bands represent useful biomarkers for the analysis of the premotor-motor network in humans and mice.

These results demonstrate strong similarities between the mouse RFA-CFA network and the human PMC-M1 network. Our results validate the mouse RFA-CFA network as a suitable model for the analysis of premotor-motor connectivity in translational research.

### Limitations

Our analysis was performed at rest and captures only the PMC-M1 (RFA-CFA) interactions at the resting state. Furthermore, we did not analyze cortical–subcortical in this study. However, this study does add detailed information on intracortical communication in the human PMC-M1 network as well as the mouse RFA-CFA network, thereby validating the mouse RFA-CFA network as a model for the human PMC-M1 network.

Our results on the RFA-CFA connectivity were performed in lightly anesthetized mice, which lead to some degree of limitations. Although our anesthesia protocol consisted of a low dose of urethane application, the influence of even this light anesthesia on our results should not be neglected and therefore a direct translation of our results to the awake state is difficult. We did, however, use anesthesia that brings conditions as close as possible to the nonanesthetized settings. A large number of in vivo electrophysiological studies have shown that urethane is the most suitable anesthetic for long recordings of spontaneous electrical activity (Maggi and Meli 1986; Sceniak and Maciver 2006; Devonshire et al. 2010; Masamoto et al. 2012). Urethane has the least impact on neuronal discharge patterns, neurotransmitter receptors, and synaptic interactions. The functional connectivity under the urethane anesthesia is very similar to the nonanesthetized state (Paasonen et al. 2018). Especially, the cortical and thalamocortical connectivity is better preserved during the urethane anesthesia as compared with other anesthetics (Paasonen et al. 2018). Since isoflurane (even at low concentrations) strongly reduces the neuronal discharge patterns, especially in the cortex, isoflurane was discontinued after transferring the mice into the recording setup (at least 45 min prior to the recording) to minimize anesthesia-induced side effects on neural activity and functional connectivity (Williams et al. 2010; Bukhari et al. 2018; Paasonen et al. 2018).

The intracortical connectivity shown here cannot be explained by volume conduction artifacts alone. This can be seen by the fact that we find the highest LFP power in the delta and theta frequency bands, while the functional and effective connectivity is strongest in the beta frequency band. This is in line with our findings in the human PMC-M1 network.

In a simulation study, Haufe et al. (2013) showed that the time reversal technique (TRT) is a suitable method to reduce the influence of weak asymmetries (e.g., noncausal interactions caused by delayed instantaneous connections [i.e., volume conduction]) on the outcome of a causal measure while preserving or even enhancing the influence of strong asymmetries (e.g., time-delayed causal interactions not caused by volume conduction). Therefore, TRT was applied as a significance test to the compounds already identified by TPDC. Accordingly, TPDC asymmetries should be insensitive to contributions from volume conduction or other instantaneous interactions. Furthermore, our TPDC asymmetry calculation should be fully reversed by applying TRT and therefore only sensitive to strong causal interactions. In another study, TPDC was shown to be insensitive to volume conduction (Joffe 2008). The control analysis was performed by testing the analyses on data where we know the ground truth through simulations. Such simulations can also help in model-based studies in electrophysiology for functional and effective connectivity. We have tested these methods extensively on simulations of autoregressive processes in previous work (Govindan et al. 2006; Muthuraman et al. 2015; Anwar et al. 2016).

In summary, our findings demonstrate a strong similarity in spontaneous activity as well as functional connectivity between the human PMC-M1 and mouse RFA-CFA. We have extensively analyzed the properties of spontaneous activity in RFA and CFA and assessed the functional and effective connectivity between both areas in lightly anesthetized wild-type mice. Performing simultaneous recordings at multiple brain regions, we demonstrate that RFA and CFA are functionally distinct cortical areas. We found that RFA and CFA are similar to human PMC and M1 and are highly interconnected and synchronized in the β- and high γ-frequency bands in a layer-specific manner. We further demonstrated that the flow of information, although bidirectional, is significantly stronger from the premotor cortex toward the primary motor cortex, both in humans and mice. Taken together, our results clearly identify the RFA-CFA network as a highly suitable mouse model for studying movement disorders in humans.

## Supplementary Material

Supplementary_material-Final-CC_bhab369

## Notes

We thank Beate Krumm for the excellent help with the histology. We thank Prof. Dr Maik Stüttgen for providing the MLIB-toolbox for the quality control of spike SUs. *Conflict of Interest:* The authors have declared that no conflict of interest exists.
