# Supplementary material for: Translational Model of Cortical Premotor-Motor Networks
Source: Cereb Cortex. 2021 Oct 23;32(12):2621–34. doi: 10.1093/cercor/bhab369 (PMC9201593; doi:10.1093/cercor/bhab369)
Supplement: Supplementary_material-Final-CC_bhab369 [file supplementary_material-final-cc_bhab369.docx]

**Supplementary material for**

**Translational model of cortical premotor-motor networks**

Svenja. L. Kreis^1*^, Heiko J. Luhmann^1*^, Dumitru Ciolac^2,3^, Sergiu Groppa^2*^, Muthuraman Muthuraman^2*^

^1^ Institute of Physiology, University Medical Center of the Johannes Gutenberg University Mainz, Mainz, Germany

^2^ Section of Movement Disorders and Neurostimulation, Biomedical statistics and multimodal signal processing unit, Department of Neurology, University Medical Center of the Johannes Gutenberg University Mainz, Mainz, Germany

^3^ Nicolae Testemitanu State University of Medicine and Pharmacy, Chisinau, Republic of Moldova.

*Equal contribution

**Supplementary Table 1.** Spectral power of spontaneous LFP activity recorded in CFA and RFA. Mean + standard deviation (n=16 mice) is shown separately for each layer and frequency band; p-values resulted from two sample t-tests. P-values < 0.017 were considered significant and are highlighted in grey

| frequency band | layer | CFA (mean ±SD) | RFA (mean ±SD) | p-value |
| --- | --- | --- | --- | --- |
| δ | l2/3 | 2.4867 ±5.4194 | 1.5323 ±5.4835 |  |
|  | l5 | 2.8941 ±8.3070 | 1.2634 ±3.6845 |  |
|  | l6 | 2.2777 ±4.8551 | 1.2119 2.5145 |  |
| θ | l2/3 | 0.6979 ±1.0824 | 0.9055 ±1.6830 |  |
|  | l5 | 0.7867 ±1.3837 | 0.4580 ±0.9375 |  |
|  | l6 | 0.7408 ±1.0973 | 0.8484 ±1.4794 |  |
| α | l2/3 | 0.0704 ±0.0906 | 0.0697 ±0.1027 |  |
|  | l5 | 0.0701 ±0.0905 | 0.0498 ±0.0772 |  |
|  | l6 | 0.0726 ±0.0866 | 0.0741 ±0.1086 |  |
| β | l2/3 | 0.0105 ±0.0067 | 0.0086 ±0.0072 |  |
|  | l5 | 0.0112 ±0.0083 | 0.0085 ±0.0059 | 0.0024 |
|  | l6 | 0.0116 ±0.0082 | 0.0101 ±0.0082 |  |
| low γ | l2/3 | 0.0045 ±0.0032 | 0.0032 ±0.0023 | 9.7975e^-04^ |
|  | l5 | 0.0045 ±0.0030 | 0.0037 ±0.0024 |  |
|  | l6 | 0.0049 ±0.0032 | 0.0040±0.0026 | 0.0410 |
| medium γ | l2/3 | 0.0010 ±0.0006 | 0.7311e^-03^ ±0.5304e^-03^ | 6.3662e^-04^ |
|  | l5 | 0.0010 ±0.0006 | 0.8348e^-03^ ±0.5221e^-03^ | 0.0090 |
|  | l6 | 0.0011 ±0.0006 | 0.8895e^-03^ ±0.5833e^-03^ | 0.0080 |
| high γ | l2/3 | 3.6413e-04±2.0489e-^04^ | 0.2825e-03 ± 0.1971e^-03^ | 0.0051 |
|  | l5 | 3.6109e-04±2.0389e^-04^ | 0.3014e-03 ±0.1967e^-03^ | 0.0171 |
|  | l6 | 4.1706e-04± 2.4914e^-04^ | 0.3028 e-03 ±0.2008e^-03^ | 0.0013 |

**Supplementary Table 2.** Number and mean firing rates of SU in RFA and CFA in different layers.

|  | RFA | | CFA | |
| --- | --- | --- | --- | --- |
|  | number of SU | SU firing rate (mean ± SD) | number of SU | SU firing rate (mean ± SD) |
| L 2/3 | 26 | 3.11 ± 4.10 | 48 | 1.97 ± 3.00 |
| L 5 | 54 | 3.83 ± 6.20 | 100 | 2.85 ± 4.29 |
| L 6 | 54 | 3.07 ± 4.10 | 154 | 3.23 ± 5.65 |
| all layers | 134 | 3.11 ± 4.67 | 302 | 2.98 ± 4.84 |

**Supplementary Table 3.** Mean ± SD of LFP coherence separated by frequency band and layer averaged across 16 mice

|  |  | **CFA L2/3** | **CFA L5** | **CFA L6** |
| --- | --- | --- | --- | --- |
| **delta** | **RFA L2/3** | 0.3814± 0.2610 | 0.4667± 0.2281 | 0.4647±0.1932 |
|  | **RFA L5** | 0.3972± 0.2407 | 0.5060± 0.2112 | 0.5641±0.1819 |
|  | **RFA L6** | 0.3749± 0.2269 | 0.4707± 0.1911 | 0.5589±0.1828 |
| **theta** | **RFA L2/3** | 0.5538± 0.2978 | 0.6300±0.2918 | 0.6472± 0.2423 |
|  | **RFA L5** | 0.6027± 0.2416 | 0.6874±0.2347 | 0.7425±0.1850 |
|  | **RFA L6** | 0.5770± 0.2108 | 0.6482±0.1870 | 0.7245±0.1426 |
| **alpha** | **RFA L2/3** | 0.5950± 0.2732 | 0.6524±0.2762 | 0.6780±0.2185 |
|  | **RFA L5** | 0.6497± 0.2062 | 0.7048± 0.2117 | 0.7473±0.1616 |
|  | **RFA L6** | 0.6134± 0.1865 | 0.6625± 0.1827 | 0.7153±0.1416 |
| **beta** | **RFA L2/3** | 0.6369±0.2367 | 0.6743±0.2286 | 0.6969±0.1726 |
|  | **RFA L5** | 0.6960± 0.1792 | 0.7309±0.1738 | 0.7670±0.1293 |
|  | **RFA L6** | 0.6858± 0.1583 | 0.7138± 0.1488 | 0.7586±0.1075 |
| **low gamma** | **RFA L2/3** | 0.6127±0.2168 | 0.6430±0.2044 | 0.6615±0.1823 |
|  | **RFA L5** | 0.6604±0.1838 | 0.6913±0.1793 | 0.7216±0.1564 |
|  | **RFA L6** | 0.6637±0.1765 | 0.6896±0.1744 | 0.7250±0.1530 |
| **medium gamma** | **RFA L2/3** | 0.4202±0.2060 | 0.4483±0.1961 | 0.4647±0.1638 |
|  | **RFA L5** | 0.4762±0.1730 | 0.5062±0.1812 | 0.5207±0.1560 |
|  | **RFA L6** | 0.4670±0.1689 | 0.4967±0.1758 | 0.5171±0.1591 |
| **high gamma** | **RFA L2/3** | 0.3037±0.1888 | 0.3264±0.1876 | 0.3249±0.1562 |
|  | **RFA L5** | 0.3554±0.1767 | 0.3760±0.1876 | 0.3717±0.1645 |
|  | **RFA L6** | 0.3228±0.1670 | 0.3400±0.1792 | 0.3473±0.1647 |

**Supplementary Table 4.** P-values resulting from LMM analysis of LFP coherence separated by layer and frequency band; - indicates not significant; p < 0.05. RFA L5 : CFA L5 indicates an interaction of the factors RFA layer and CFA layer on the resulting coherence.

|  | **δ** | **θ** | **α** | **β** | **low γ** | **medium γ** | **high γ** |
| --- | --- | --- | --- | --- | --- | --- | --- |
| **RFA L5** | - | 6.017e^-04^ | 2.447e^-05^ | 3.851e^-08^ | 1.715e^-05^ | 2.155e^-07^ | 1.627e^-06^ |
| **RFA L6** | - | - | - | 9.519e^-05^ | 8.082e^-05^ | 1.992e^-04^ | - |
| **CFA L5** | 1.285e^-10^ | 1.265e^-07^ | 1.201e^-05^ | 5.752e^-04^ | 6.816e^-03^ | 0.010 | 0,037 |
| **CFA L6** | 1.018e^-09^ | 3.172e^-10^ | 8.022e^-10^ | 8.061e^-08^ | 2.419e^-05^ | 7.418e^-05^ | 5.802e^-0.2^ |
| **RFA L5 : CFA L5** | - | - | - | - | - | - | - |
| **RFA L6 : CFA L5** | - | - | - | - | - | - | - |
| **RFA L5 : CFA L6** | 2.995e^-06^ | 0.017 | - | - | - | - | - |
| **RFA L6 : CFA L6** | 1.170e^-06^ | 0.016 | - | - | - | - | - |

**Supplementary Table 5.** TPDC results, separated by layer combination, direction and frequency band, mean + standard deviation averaged across n=16 mice: p-values correspond to two-sample t-tests.

| frequency | layer combination | RFA towards CFA | CFA towards RFA | p |
| --- | --- | --- | --- | --- |
| δ | CFA 2/3-RFA 2/3 | 0.6852±0.1458 | 0.5904±0.1472 |  |
|  | CFA 5-RFA 2/3 | 0.6225±0.1459 | 0.5904±0.1472 |  |
|  | CFA 6-RFA 2/3 | 0.5960±0.1264 | 0.6189±0.1152 |  |
|  | CFA 23-RFA 5 | 0.6756±0.1718 | 0.5358±0.1583 | 0.0231 |
|  | CFA 5-RFA 5 | 0.6300±0.1514 | 0.6049±0.1425 |  |
|  | CFA 6-RFA 5 | 0.6092±0.1540 | 0.6229±0.1359 |  |
|  | CFA 23-RFA 6 | 0.6685±0.1235 | 0.5456±0.1317 | 0.0107 |
|  | CFA 5-RFA 6 | 0.6195±0.1310 | 0.6099±0.1473 |  |
|  | CFA 6-RFA 6 | 0.6087±0.1012 | 0.6321±0.0870 |  |
| θ | CFA 2/3-RFA 2/3 | 0.6616±0.1291 | 0.5405±0.1497 | 0.0203 |
|  | CFA 5-RFA 2/3 | 0.6328±0.1341 | 0.5931±0.1560 |  |
|  | CFA 6-RFA 2/3 | 0.6148±0.1084 | 0.6147±0.1060 |  |
|  | CFA 2/3-RFA 5 | 0.6700±0.1595 | 0.5465±0.1534 | 0.0332 |
|  | CFA 5-RFA 5 | 0.6494±0.1380 | 0.5970±0.1364 |  |
|  | CFA 6-RFA 5 | 0.6241±0.1491 | 0.6246±0.12/35 |  |
|  | CFA2/3-RFA 6 | 0.6717±0.1046 | 0.5564±0.1293 | 0.0095 |
|  | CFA 5-RFA 6 | 0.6436±0.1150 | 0.6027±0.1343 |  |
|  | CFA 6-RFA 6 | 0.6247±0.0889 | 0.6340±0.0772 |  |
| α | CFA2/3-RFA 2/3 | 0.6437±0.1296 | 0.5273±0.1426 | 0.0220 |
|  | CFA 5-RFA 2/3 | 0.6092±0.1327 | 0.5763±0.1501 |  |
|  | CFA 6-RFA 2/3 | 0.5907±0.1141 | 0.5957±0.1011 |  |
|  | CFA2/3-RFA 5 | 0.6494±0.1627 | 0.5384±0.1565 |  |
|  | CFA 5-RFA 5 | 0.6267±0.1403 | 0.5904±0.1421 |  |
|  | CFA 6-RFA 5 | 0.6056±0.1489 | 0.6135±0.1248 |  |
|  | CFA2/3-RFA 6 | 0.6487±0.1070 | 0.5464±0.1271 | 0.0198 |
|  | CFA 5-RFA 6 | 0.6185±0.1137 | 0.5984±0.1342 |  |
|  | CFA 6-RFA 6 | 0.6046±0.0867 | 0.6279±0.0736 |  |
| β | CFA2/3-RFA 2/3 | 0.6528±0.1291 | 0.5401±0.1368 | 0.0230 |
|  | CFA 5-RFA 2/3 | 0.6167±0.1354 | 0.5897±0.1507 |  |
|  | CFA 6-RFA 2/3 | 0.5959±0.1251 | 0.5959±0.1251 |  |
|  | CFA2/3-RFA 5 | 0.6554±0.1645 | 0.5485±0.1530 |  |
|  | CFA 5-RFA 5 | 0.6305±0.1408 | 0.6037±0.1340 |  |
|  | CFA 6-RFA 5 | 0.6092±0.1514 | 0.6037±0.1340 |  |
|  | CFA2/3-RFA 6 | 0.6568±0.1103 | 0.5547±0.1298 | 0.0229 |
|  | CFA 5-RFA 6 | 0.6254±0.1125 | 0.6091±0.1343 |  |
|  | CFA 6-RFA 6 | 0.6134±0.0869 | 0.6403±0.0667 |  |
| low γ | CFA2/3-RFA 2/3 | 0.6613±0.1251 | 0.5379±0.1354 |  |
|  | CFA 5-RFA 2/3 | 0.6222±0.1333 | 0.5891±0.1499 |  |
|  | CFA 6-RFA 2/3 | 0.6003±0.1312 | 0.6104±0.0939 |  |
|  | CFA2/3-RFA 5 | 0.6595±0.1602 | 0.5450±0.1518 | 0.0465 |
|  | CFA 5-RFA 5 | 0.6315±0.1373 | 0.6045±0.1323 |  |
|  | CFA 6-RFA 5 | 0.6069±0.1539 | 0.6304±0.1091 |  |
|  | CFA2/3-RFA 6 | 0.6641±0.1088 | 0.5503±0.1396 | 0.0153 |
|  | CFA 5-RFA 6 | 0.6298±0.1087 | 0.6090±0.1423 |  |
|  | CFA 6-RFA 6 | 0.6145±0.0846 | 0.6407±0.0631 |  |
| medium γ | CFA2/3-RFA 2/3 | 0.6462±0.1227 | 0.5375±0.1458 |  |
|  | CFA 5-RFA 2/3 | 0.6026±0.1329 | 0.5883±0.1450 |  |
|  | CFA 6-RFA 2/3 | 0.5839±0.1246 | 0.6085±0.0889 |  |
|  | CFA2/3-RFA 5 | 0.6513±0.1536 | 0.5352±0.1523 | 0.0399 |
|  | CFA 5-RFA 5 | 0.6192±0.1368 | 0.5958±0.1266 |  |
|  | CFA 6-RFA 5 | 0.5952±0.1484 | 0.6192±0.1025 |  |
|  | CFA2/3-RFA 6 | 0.6540±0.1010 | 0.5431±0.1401 | 0.0154 |
|  | CFA 5-RFA 6 | 0.6139±0.1080 | 0.6010±0.1382 |  |
|  | CFA 6-RFA 6 | 0.5994±0.0785 | 0.6325±0.0584 |  |
| high γ | CFA2/3-RFA 2/3 | 0.6206±0.1072 | 0.5104±0.1368 | 0.0166 |
|  | CFA 5-RFA 2/3 | 0.5776±0.1234 | 0.5563±0.1404 |  |
|  | CFA 6-RFA 2/3 | 0.5633±0.1078 | 0.5758±0.0818 |  |
|  | CFA2/3-RFA 5 | 0.6188±0.1509 | 0.5050±0.1469 | 0.0388 |
|  | CFA 5-RFA 5 | 0.5867±0.1342 | 0.5615±0.1208 |  |
|  | CFA 6-RFA 5 | 0.5633±0.1417 | 0.5815±0.0985 |  |
|  | CFA2/3-RFA 6 | 0.6182±0.0996 | 0.5149±0.1267 | 0.0157 |
|  | CFA 5-RFA 6 | 0.5800±0.1056 | 0.5673±0.1285 |  |
|  | CFA 6-RFA 6 | 0.5669±0.0746 | 0.5938±0.0573 |  |
